# Poly (Methyl Methacrylate)-Containing Silver-Phosphate Glass Exhibits Potent Antimicrobial Activity without Deteriorating the Mechanical and Biological Properties of Dental Prostheses

**DOI:** 10.3390/polym15020297

**Published:** 2023-01-06

**Authors:** Song-Yi Yang, Myung-Jin Lee

**Affiliations:** 1 Department of Dental Hygiene, Konyang University, Daejeon 35365, Republic of Korea; 2 Department of Dental Hygiene, Division of Health Science, Baekseok University, Cheonan 31065, Republic of Korea

**Keywords:** antimicrobial effect, dental materials, silver-phosphate glass, poly (methyl methacrylate)

## Abstract

Poly (methyl methacrylate) (PMMA) is a commonly used denture material with poor antimicrobial effects. This study investigated the antimicrobial effects of PMMA-containing silver-phosphate glass. We fabricated a novel material comprising PMMA-containing silver-phosphate glass. Then, microhardness, flexural strength, and gloss unit were analyzed. Antimicrobial activity against *Streptococcus mutans* and *Candida albicans* was investigated. Colony-forming units were counted, and antimicrobial rates were measured. Biocompatibility tests were performed using a colorimetric MTT assay for evaluating cell metabolic activity. The microhardness, flexural strength, and gloss unit of the experimental groups (with silver-phosphate glass) were not significantly different from those of the control group (no silver-phosphate glass) (P > 0.05), which showed clinically valid values. With increasing proportions of silver-phosphate glass, the antimicrobial activity against the two microorganisms increased (P < 0.05). Furthermore, *S. mutans* showed more than 50% antimicrobial activity in 4%, 6%, and 8% experimental groups, *C. albicans* showed more than 50% antimicrobial activity in 6% and 8% groups, and a statistically significant difference in antimicrobial activity was observed compared to the control (P < 0.05). The cell viability of the experimental groups was not significantly different from that of the control group (P > 0.05). Both control and experimental groups showed approximately 100% cell viability. These results suggest that silver-phosphate glass is a promising antimicrobial material in dentistry.

## 1. Introduction

Various polymers are used in dental treatment; they are widely used as aesthetic and prosthetic restoration materials and cement [1,2]. In particular, poly (methyl methacrylate) (PMMA), which was introduced in dentistry a long time ago, is used in various applications, from fixed prostheses to removable prostheses [3,4]. The choice of polymers used as dental materials depends on their application. Moreover, with the development of a new dental prosthesis, a suitable polymer is also developed [5]. Although PMMA is the most widely used material for denture bases because of its good aesthetics, low absorption and solubility, and nontoxicity to the human body, it has the disadvantage of having poor antimicrobial properties [1,6,7]. For materials intended for dental applications, it is important to consider the adhesion of various microorganisms present in the oral cavity to the material [8,9]. Furthermore, a large portion of a dental prostheses is in contact with the mucous membrane; therefore, it is necessary to consider *Candida albicans*, the main causative agent of denture stomatitis, in patients using removable prostheses [8,10]. *C. albicans* is a fungus that adheres well to PMMA and other polymers used in dental implants [9,11]. The adhesion of *C. albicans* to the prostheses causes a biofilm formation in the early stage and leads to denture stomatitis. The adhesion is affected by various factors, such as the roughness of the dental material, surface energy, and the degree of hydrophobicity of the dental material surface [10]. *Streptococcus mutans,* present in the oral cavity, is the main causative agent of denture stomatitis; therefore, it is important to consider developing an antimicrobial material that acts against it [12,13]. Generally, *S. mutans* has a greater ability to form biofilms than other oral bacterial species [14]. 

*S. mutans* has a relatively high recurrence rate, because of its low sensitivity to antibiotics and low compliance to treatment in elderly patients [9,15]. Therefore, it is important to prevent the onset of denture stomatitis caused by these two microbes. The development of antimicrobial PMMA materials is necessary to achieve this goal [16].

In previous studies, silver, which is known to have antibacterial properties, has been used to make new dental materials [16,17]. Silver nanoparticles (AgNPs) exert a strong antibacterial effect by maximizing the surface area of silver; however, they have the disadvantage of inducing high silver toxicity, weakening the mechanical properties of the material, and the antibacterial effect was only short term [8,18]. In addition, the use of graphene oxide was studied [15]. Although it shows an antibacterial effect by forming hydroxyl radicals or reactive oxygen species on the graphene surface, its black color has limited its use in aesthetic dental materials [15].

Recently, a method for stably binding metal ions and slowly dissolving them over time to induce an antimicrobial effect has been developed, and studies using this method on glass are being actively conducted [18,19]. The addition of antimicrobial substances to glass has an inhibitory effect on bacterial and fungal growth [7]. Phosphate-based glass controls the dissolution rate of the phosphate ions by slowly releasing the ions over a long period [20]. Silver-phosphate glass is a novel porous glass designed to combine bioactive and antimicrobial properties; this is a method to stably support metal ions that act as antimicrobial agents and to have a continuous effect with a slow release of the ions [18,20,21]. Based on this method, we fabricated a sustainable antibacterial glass containing Ag ions bound to phosphate-based glass. Furthermore, we attempted to construct a PMMA-containing silver-phosphate glass to prevent denture stomatitis caused by oral microorganisms.

Therefore, this study aimed to examine the effect of adding silver-phosphate glass to PMMA and analyze its surface and mechanical properties, antimicrobial effects, and biocompatibility. The null hypotheses of this study are that (1) PMMA containing silver-phosphate glass does not result in significant differences in the surface and mechanical properties compared to the control, (2) PMMA containing silver-phosphate glass does not result in significant differences in the antimicrobial properties compared to the control, and (3) PMMA containing silver-phosphate glass does not result in significant differences in biocompatibility compared to the control.

## 2. Materials and Methods

### 2.1. Preparation and Characterization of Silver-Phosphate Glass

To prepare the silver-phosphate glass, P_2_O_5_ (65 mol.%), CaO (10 mol.%), Na_2_O (23 mol.%), and Ag_2_O (2 mol.%) powders were mixed for 60 min, and the mixture was melted in an alumina crucible at 1200 °C for 60 min. The melted glass was quenched to obtain glass. Subsequently, the glass was ground in an alumina mortar and pulverized under dry conditions using a planetary mono mill (Pulverisette-7; Fritsch, Idar-Pberstein, Germany) [19]. X-ray diffraction (XRD) was used to confirm the silver-phosphate glass phase. The XRD (Ultima IV, Rigaku, Tokyo, Japan) measurements were performed to analyze the crystal structure of the manufactured glass. A 2θ angle range of 10° to 80° was used with a scanning rate of 2°/min.

### 2.2. Fabrication of PMMA Containing Silver-Phosphate Glass

Commercially available PMMA (Probase Cold, Ivoclar Vivadent, Germany) was used in this study. The silver-phosphate glass powder was mixed with the acrylic resin powder, the weight percentages were calculated to match the final concentration in the resin at various weight concentrations (0, 2, 4, 6, and 8 wt. %). PMMA without silver-phosphate glass was used as the control. The silver-phosphate glass powder was mixed until homogenous with the PMMA using a high-speed mixer (Speed Mixer, Hauschild, Hamm, Germany) at 3500 rpm for 2 min. The zeta-potential of silver-phosphate glass, suspended in DW (1 mg/mL), were measured using Zetasizer Nano-zs90 (Malvern Instruments Ltd., Malvern, UK). To further evaluate their stability, 1 mg of HINPs was dispersed in PBS at pH 5.5, optimized with HCl and NaOH. The compositions of the control and experimental groups are summarized in Table 1. 

### 2.3. Scanning Electron Microscopy Surface Images

To examine the surface of specimens, the control and silver-phosphate glass groups were attached to metal stubs via double adhesive carbon tape, sputter coated with gold in a vacuum evaporator for 100 s at 20 mA, and they were examined using field-emission scanning electron microscopy (FE-SEM; JEOL-7800F, Tokyo, Japan) operated at an acceleration voltage of 1.0 kV at a magnification of 2000×.

### 2.4. Microhardness Analysis

Vickers hardness tests were performed using a Micro Vickers hardness tester (DMH-2; Matsuzawa Skik, Japan) with a diamond pyramid indenter. The face angle of the indenter was set at 136°. The Vickers hardness was measured with the application of a 300 g load for 30 s. Before each test, calibration was conducted for each specimen. The average value among three different points was determined as the final value of Vickers hardness.

### 2.5. Flexural Strength

The mechanical properties of the PMMA-silver-phosphate glass were measured according to ISO 20795-2 [13]. Each group consisted of ten samples of the fabricated material, with a dimension of 3.3 × 10 × 25 mm. A computer-controlled universal testing machine (Model 3366; Instron^®^, Norwood, MA, USA) was used to fracture the specimens in a three-point flexure. The flexural strength and elastic modulus were measured at a span length of 50 mm and a crosshead speed of 5 mm/min. The flexural strength was calculated as follows:

σ = 3Fl /(2bh^2^), (1)

where F denotes the maximum load, l denotes the distance, b denotes the width, and h denotes the height.

The elastic modulus was calculated as follows:

E = F1l^3^/(4bh^3^d), (2)

where F1 is the load at a point in the straight-line portion of the stress–strain graph, d is the deflection at load F1, l is the distance, b is the width, and h is the height. 

### 2.6. Surface Gloss Analysis

Disc-shaped samples with a diameter of 15 mm and thickness of 2 mm (*n* = 10) were used to measure surface gloss at an incident angle of 60° using a calibrated infrared glossmeter (IG 330; Horiba Ltd., Kyoto, Japan). An average of six measurements was recorded for each surface.

### 2.7. Antimicrobial Properties 

We examined the antimicrobial activities of *Streptococcus mutans* (ATCC 25175) and *Candida albicans* (ATCC 10231). *S. mutans* was cultured in a brain heart infusion medium (Becton Dickinson and Co., MD, USA), and *C. albicans* was cultured in yeast mold (YM, Becton Dickinson and Co., Franklin Lakes, NJ, USA). Both microorganisms were incubated at 37 °C for 24 h. To investigate the antimicrobial properties of the PMMA-silver-phosphate glass samples, 1 mL (1 × 108 cells/mL) of each microbial sample was placed on the PMMA samples and incubated at 37 °C for 24 h. The PMMA samples were rinsed with distilled water to remove non-attached microorganisms, and the attached microorganisms were detached using ultrasonication for 3 min (Ultrasonic Cleaner SH-2100; Saehan Ultrasonic). The samples were serially diluted and spread onto a solid agar plate and incubated at 37 °C for 24 h. Subsequently, the colony-forming units (CFUs) were estimated. The antimicrobial rates were calculated using the following equation [14]:

Antimicrobial rate = N_0_ − N_x_/N_0_ × 100%, (3)

where N_0_ is the CFU number of the blank control group (microorganisms cultured in culture medium), and N_x_ is the CFU number of the experimental groups (x = 0%, 2%, 4%, 6%, or 8% experimental groups).

### 2.8. Biocompatibility

An MTT (3-(4,5-dimethylthiazol-2-yl)-2,5-diphenyl tetrazolium bromide) assay was carried out to investigate the cell viability of the control and experimental groups, according to the international guideline; ISO 10993-5.

First, the experimental and control samples were soaked in cell culture media (RPMI 1640; Gibco, Grand Island, NY, USA) for 24 h at 37 °C. The extraction method was carried out according to ISO 10993-12 (Biological evaluation of medical devices—Part 12: Sample preparation and reference materials) [9]. L929 cells (mouse fibroblasts, NCTC clone 929; Korean Cell Line Bank, Korea) were cultured on 96-well plates (SPL, Pocheon-si, Gyeonggi-do, Republic of Korea) at a density of 1 × 10^5^ cells/mL (100 µL of culture medium) for 24 h. The culture supernatants were discarded, and 100% extractions at 0.2 g/mL of the control and experimental samples were added to each well. The cultures were maintained for 24 h, and then the culture media were discarded and replaced with 50 µL of thiazolyl blue tetrazolium bromide (MTT; Sigma, St. Louis, MO, USA) solution. Following 2 h of incubation, the MTT solution was discarded, and 100 µL of isopropanol (Sigma, St. Louis, MO, USA) was added to each well. Absorbance was measured spectrophotometrically at 570 nm using an ELISA reader (Epoch; BioTek, Winnoski, VT, USA).

### 2.9. Statistical Analysis

All statistical analyses were performed using the IBM SPSS software (version 23.0; IBM Korea Inc., Seoul, Republic of Korea) for Windows, using data from at least three independent experiments. The results obtained from the control and experimental groups were analyzed using a one-way analysis of variance (ANOVA) followed by Tukey’s test. Statistical significance was set at P < 0.05.

## 3. Results 

### 3.1. Characterization of the Silver-Phosphate Glass

**Figure 1:**
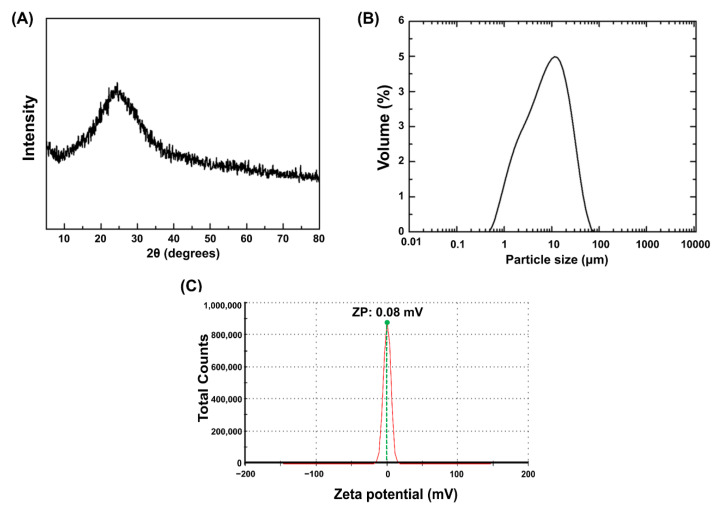
Particulate properties of silver-phosphate glass: (**A**) X-ray diffraction patterns of silver-phosphate glass, (**B**) particle size distribution, (**C**) zeta potential distribution.

The XRD patterns of silver-phosphate glass are shown in Figure 1A. The absence of sharp peaks indicates the general non-crystalline nature of glass, confirming that the fabricated silver-phosphate glass exhibited characteristics of an amorphous glass. Figure 1B shows the particle size distribution of the silver-phosphate glass; particle size ranges from 0.9 to 28 μm with a median diameter (d50) of 7.4 μm. As shown in Figure 1C, the silver-phosphate glass has a zeta potential value of 0.08 mV. Therefore, the isoelectric point (IEP, ~0mV) of silver-phosphate glass is approximately at pH 5.5, and it has a slightly higher positive zeta potential around pH 5.5. 

### 3.2. SEM Micrographs

**Figure 2:**
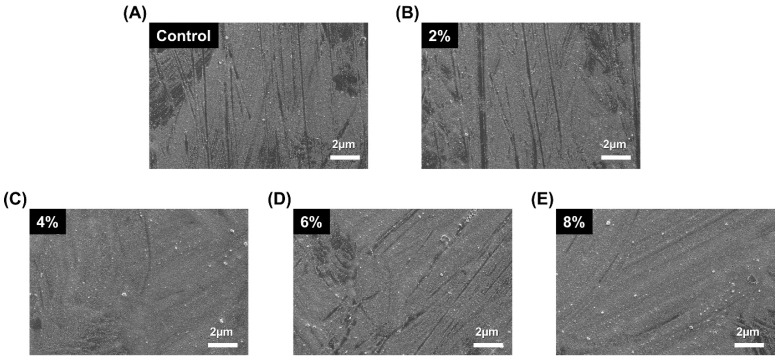
Representative SEM micrographs of the sample surfaces: (**A**) control, (**B**) 2%, (**C**) 4%, (**D**) 6%, (**E**) 8%.

As shown in Figure 2, the SEM micrographs of the sample surfaces indicated no significant differences between each group of increasing silver-phosphate composition in the surface morphology. 

### 3.3. Microhardness Analysis

**Figure 3:**
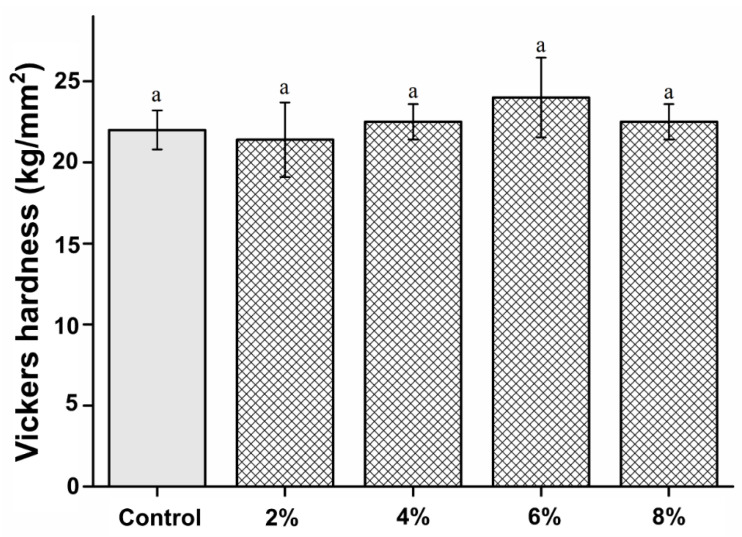
Comparison of Vickers hardness between the samples. “a” indicates that there is no statistically significant difference between the groups (P > 0.05).

The microhardness of the PMMA-silver-phosphate glass in the experimental groups was not significantly different from that of the control group (P > 0.05). The microhardness results are shown in Figure 3. The microhardness values of the control, 2%, 4%, 6%, and 8% groups are 22.22 ± 1.21, 21.41 ± 2.32, 22.51 ± 1.13, 24.24 ± 2.41, and 22.51 ± 1.12 kg/mm^2^, respectively. 

### 3.4. Flexural Strength

**Figure 4:**
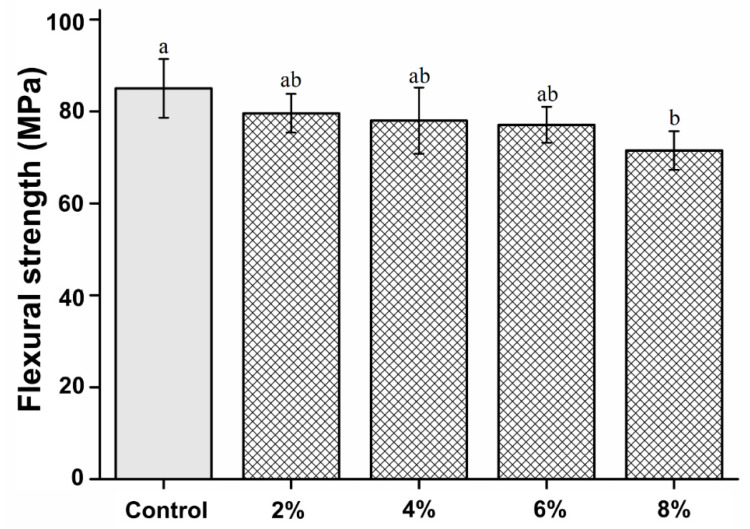
Comparison of the flexural strength between the groups. Different letters indicate a statistically significant difference (P < 0.05).

The flexural strength of the experimental groups was not significantly different from that of the control (P > 0.05), except for the 8% experimental group, which had a significantly lower flexural strength than the control group (P < 0.05). The flexural strength results are shown in Figure 4. Nevertheless, no statistically significant differences were observed between the experimental groups (P > 0.05).

### 3.5. Surface Gloss

**Figure 5:**
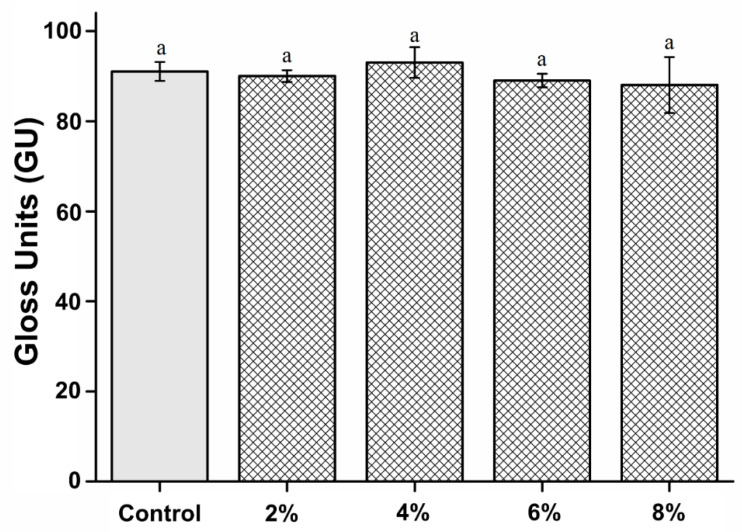
Comparison of surface gloss between the groups. “a” indicates that there is no statistically significant difference (P > 0.05).

The surface gloss of the experimental groups was not significantly different from that of the control group (P > 0.05). The microhardness results are shown in Figure 5. The microhardness values of the control, 2%, 4%, 6%, and 8% groups are 91.34 ± 2.11, 90.25 ± 1.35, 93.01 ± 3.44, 89.57 ± 1.56, and 88.27 ± 6.25 gloss units, respectively. 

### 3.6. Antimicrobial Properties

**Figure 6:**
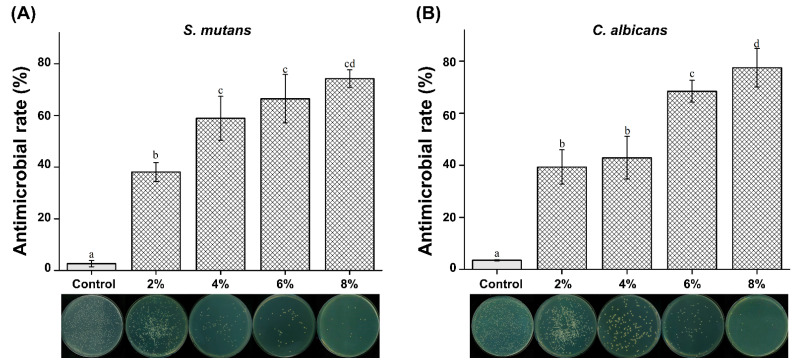
Antimicrobial rates of (**A**) *S. mutans* and (**B**) *C. albicans*. The same letter indicates no statistically significant difference (P > 0.05). Different letters indicate a statistically significant difference (P < 0.05).

We found that all concentrations of silver-phosphate glass reduced the CFU for both *S. mutans* and *C. albicans*. The antimicrobial rates are shown in Figure 6. The antimicrobial rates of both *S. mutans* and *C. albicans* were significantly increased in the 2, 4, 6, and 8% groups compared to the control group (P < 0.05). The antimicrobial activity increased with increasing concentration of silver-phosphate in the PMMA. 

### 3.7. Biocompatibility Analysis

**Figure 7:**
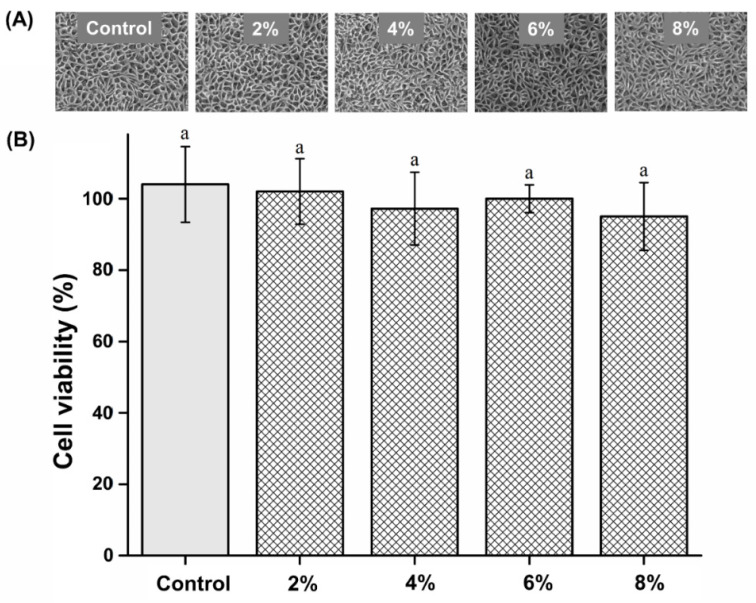
(**A**) Micrographs of L929 cells. (**B**) Comparison of cell viability of the different groups. “a” indicates no statistically significant difference (P > 0.05).

Cell viability of the experimental groups was not significantly different from that of the control group (P > 0.05). The cell viability results are shown in Figure 7. The cell viability of the control, 2%, 4%, 6%, and 8% groups are 104.03 ± 10.62, 102.35 ± 9.27, 97.28 ± 10.29, 100.24 ± 3.99, and 95.47 ± 9.57%, respectively. 

## 4. Discussion

In this study, PMMA-containing silver-phosphate glass was successfully developed and showed excellent antimicrobial activity with no negative influence on the materials’ mechanical strength, surface gloss, and biocompatibility.

The analysis of the physical properties of dentures is essential for clinical application [2,22]. In this study, PMMA, a well-known pro-base resin used in dentistry for its antimicrobial properties, was optimized. A novel PMMA with silver-phosphate glass was successfully developed.

Among the physical properties, the surface hardness of the PMMA-silver-phosphate material was evaluated because it can be used to measure the wear resistance in a non-destructive manner [22,23]. Moreover, the surface hardness of a material is correlated with its mechanical strength [24] and can be used to indicate the maximum force that the denture base resin will resist during mastication [5,22].

Herein, we found that the PMMA-silver-phosphate glass material had a similar surface hardness to PMMA, showing that the silver-phosphate glass did not affect the surface hardness. This implies that this modified version of denture base resin can be used continuously [1,4]. Denture fractures often occur in clinical practice and are closely related to the flexural strength of the resin [4,5]. Flexural strength refers to the strength that an object can withstand [22,24]. Therefore, it was imperative to measure the flexural strength of the modified PMMA-silver-phosphate glass material [2]. Notably, the denture specimens manufactured in this study were according to international standards, and their flexural strengths were measured. There was no statistically significant difference in the bending strength of the 2%, 4%, and 6% experimental groups compared with that of the control group. However, the 8% experimental group showed significantly reduced bending strength compared with that of the control group. According to the international standard for denture base resins, the three-point bending strength should be at least 65 MPa [25]. All experimental groups, except the 8% and the control groups, showed a flexural strength of at least 65 MPa. Therefore, the 2%, 4%, and 6% group compositions can be used for clinical applications [24,25]. 

Owing to aesthetic reasons, the surface gloss of denture base resins is essential for dental applications [26]. Gloss is an optical phenomenon expressed as the amount of light reflected from a surface [26,27]. Therefore, the reflectance varies according to the angle of incidence on the surface, and the glossiness differs depending on the degree of surface finish [28]. However, the oral environment provides a poor environment for maintaining the surface gloss of denture resins because they are exposed to repeated loads and high stress. Additionally, denture resins are always soaked in saliva and experience a wide range of temperature changes, greatly affecting the resin matrix and reducing the gloss [9]. Therefore, to ensure the aesthetic and longevity of the denture base resin, its surface must be smooth and glossy [27]. In this study, there was no statistically significant difference in the glossiness between the 2%, 4%, 6%, and 8% experimental groups and the control group. This shows that the PMMA-silver-phosphate glass-based denture resins are similar to commercially available PMMA resins. Thus, our first null hypothesis stating “PMMA containing silver-phosphate glass would not result in significant differences in the surface and mechanical properties compared to the control” is accepted

Denture base materials are exposed to the oral microflora, and because they are not antibacterial, they must always be kept clean [6,8,29]. Cleaning methodology is divided into mechanical and chemical methods [27,29]. The mechanical method comprises toothbrushing with toothpaste or an ultrasonic cleaner, and the chemical method includes the use of a denture cleaner and the use of a disinfectant [3]. However, according to previous studies, it is difficult to completely clean the denture using a mechanical method, and the surface of the denture base usually gets damaged owing to an incorrect cleaning method [29]. Moreover, denture cleansers can harm the eyes or skin and affect the physical strength and surface shape of the denture base resin [30,31]. Because of these shortcomings, it is necessary to develop antibacterial denture base resins to rely less on cleaning. The antimicrobial activities of the PMMA-silver-phosphate glass denture against *S. mutans* and *C. albicans* were evaluated in this study. The oral microflora comprises bacteria, viruses, and fungi, with approximately 500 types of bacteria [32,33]. In particular, *S. mutans* is the main pathogen that forms oral biofilms [19], and *C. albicans* is a fungus that causes opportunistic infections, including candidiasis [6,15,16]. In addition, *C. albicans* has developed antifungal resistance due to the use of antifungal agents; it is necessary to be careful about candida infection [34]. 

The results of this study’s antimicrobial activity test showed antimicrobial rates of less than 50% in the 2% group for *S. mutans* and the 2% and 4% groups for *C. albicans*. Although we cannot construe high antimicrobial activity for all experimental groups, there was a statistically significant difference in the 4% and 6% groups compared with the 2% groups. There was no statistically significant difference between the 4% and 6% groups. The 8% experimental group showed the highest antibacterial rate. For *C. albicans*, the antimicrobial rate was significantly increased in all experimental groups compared to that in the control group. There was no significant difference between the 2% and 4% groups, but there was a significant increase in the 6% group. The 8% experimental group showed the highest antimicrobial rate. Thus, our second null hypothesis stating “PMMA containing silver-phosphate glass would not result in significant differences in the antimicrobial properties compared to the control” is rejected. Antimicrobial elements, such as silver, copper, and zinc, are added to glass to inhibit the growth of bacteria and fungi [18]. Phosphate-based glass has been applied to biomaterials, such as artificial bones and teeth [19]. Additionally, it is an environmentally friendly material. Silver-phosphate glass stably supports metal ions and exhibits continuous antimicrobial activity with a slow elution rate [19,20].

Silver disrupts bacterial cell membranes and the functions of crucial metabolic proteins and enzymes [17]. Phosphate glass can be used as a carrier for silver ions [7,19]. Previous studies demonstrate that silver-phosphate glass has bactericidal properties against various pathogens, such as *Staphylococcus epidermidis*, *Enterococcus faecalis*, and *Streptococcus mutans* [18,21]. Several studies have attributed the antimicrobial action of silver-phosphate glass exclusively to the leaching of silver ions from the glass matrix [18,21]. Other studies have shown that the bactericidal effect of silver-phosphate glass results from the high-rate release of silver ions, and it is not cytotoxic [17,35]. We evaluated the cytotoxicity of the novel PMMA-silver-phosphate glass using the MTT assay. When selecting a biocompatibility test for a medical device, the MTT assay is recommended [6]. Cell viability was assessed by applying the extractions of the control and experimental samples to L929 cells according to the international standard for biocompatibility evaluation, namely MTT analysis [6,9]. Because the denture base is in direct contact with the oral mucosa, it is essential to use a biocompatible material that does not cause hypersensitivity or toxicity [30,36,37,38]. Based on the findings of the MTT assay, there was no statistically significant difference in cell viability between the experimental and control groups. This shows that PMMA containing the silver-phosphate glass was not cytotoxic. Thus, our third null hypothesis stating “PMMA containing silver-phosphate glass would not result in significant differences in biocompatibility compared to the control” is accepted. 

Silver-phosphate glass has promising applications in preventing and treating oral diseases because of its biocompatibility and excellent antimicrobial effect against oral pathogens. This study was conducted using only two types of microorganisms that cause oral diseases, namely *S. mutans* and *C. albicans*. They have also been used in previous studies to confirm the antimicrobial effects of newly developed dental materials [12,19]. However, it is necessary to consider a wider spectrum of microorganisms and clinical isolates to test the antimicrobial effects of a new dental material. In further study, the antimicrobial effect of silver-phosphate glass should be validated through comparative studies with a standard antimicrobial agent. In addition, this study has the limitation of carrying out the in vitro tests over a short period of time. Long-term observations and in vivo experiments are needed for future studies [39].

## 5. Conclusions

In the present study, we successfully prepared silver-phosphate glass and incorporated it into PMMA. The silver-phosphate glass did not affect the microhardness and flexural strength or the gloss unit of PMMA. Moreover, it had increased antimicrobial activity and was biocompatible. These results suggest that this novel PMMA-silver-phosphate glass material is promising for various dental applications. 

## Figures and Tables

**Table 1:** Composition of groups.

Group	Group Code	PMMA, wt%	Silver-Phosphate Glass, wt%
1	Control	100	0
2	2%	98	2
3	4%	96	4
4	6%	94	6
5	8%	92	8

## Data Availability

Not applicable.
